# Cancer-Related Cognitive Impairment in Breast Cancer: Current State of Knowledge, Mechanisms, Diagnosis, Prevention and Treatment

**DOI:** 10.3390/cancers18121974

**Published:** 2026-06-17

**Authors:** Federica Andreis, Chiara Deori, Valentina Giubileo, Chiara Abeni, Irene Caramella, Sara Cherri, Brunella Di Biasi, Michela Libertini, Silvia Noventa, Chiara Ogliosi, Ester Oneda, Tiziana Prochilo, Fausto Angelo Meriggi, Alberto Zaniboni

**Affiliations:** Oncology Department, Istituto Ospedaliero Fondazione Poliambulanza, via Bissolati 57, 25124 Brescia, Italyfausto.meriggi@poliambulanza.it (F.A.M.);

**Keywords:** breast cancer, cancer-related cognitive impairment, chemobrain, neuroinflammation, cognitive rehabilitation, physical exercise, mindfulness-based interventions, quality of life

## Abstract

Cancer-related cognitive impairment (CRCI), also known as chemobrain or chemofog, is characterized by subjective and/or objective changes in attention, executive functions, memory, and processing speed in patients with non-CNS cancers, particularly women with breast cancer. This structured narrative review synthesizes current evidence on mechanisms, neuropsychological assessment, neuroimaging correlates, clinical and demographic risk factors, emerging artificial intelligence and machine learning applications, and non-pharmacological approaches to CRCI in breast cancer. A structured literature search was conducted using PubMed/MEDLINE, PsycInfo, and Clinical Key up to May 2026, with emphasis on studies published between 2023 and 2026. Peer-reviewed English-language studies involving adult breast cancer populations and addressing predefined thematic domains of CRCI were considered. Given the heterogeneity of study designs, assessment tools, interventions, and outcomes, the findings were synthesized narratively. Current evidence supports a multifactorial model of CRCI involving neurobiological, treatment-related, psychological, and behavioral mechanisms. Neuroinflammation, endocrine disruption, oxidative stress, glial alterations, and structural or functional brain changes may contribute to cognitive symptoms; however, the strength of evidence varies, and many findings remain correlational or preclinical. Non-pharmacological interventions, including cognitive training, physical activity, mindfulness-based and psychological approaches, and multimodal digital programs, appear promising as supportive strategies. However, evidence remains heterogeneous, with benefits more consistently reported for patient-reported outcomes, fatigue, emotional distress, and quality of life than for objective neuropsychological performance. CRCI in breast cancer should be approached as a heterogeneous condition requiring early recognition, standardized assessment, and multidisciplinary supportive care. Future research should prioritize longitudinal designs, harmonized endpoints, and a clearer distinction between subjective and objective outcomes.

## Simple Summary

Cancer-related cognitive impairment (CRCI), often referred to as “chemobrain” or “chemofog”, is a frequent concern among women treated for breast cancer. It may involve difficulties with attention, memory, executive functions, and processing speed and can affect daily life, functioning at work, emotional well-being, and quality of life. This structured narrative review summarizes recent evidence on CRCI in breast cancer, focusing on possible biological mechanisms, neuropsychological assessment, neuroimaging findings, clinical and demographic risk factors, artificial intelligence and machine learning applications, and non-pharmacological supportive strategies. Current evidence suggests that CRCI is a multifactorial condition influenced by cancer treatments, inflammation, hormonal changes, fatigue, sleep disturbances, emotional distress, and individual vulnerability. Neuroimaging and biomarker studies present important research insights, but they are not yet ready for routine clinical diagnosis. Cognitive training, physical activity, mindfulness-based and psychological interventions, and digital multimodal programs appear promising, particularly for addressing perceived cognitive difficulties, fatigue, distress, and quality of life. However, evidence remains heterogeneous, and improvements in objective cognitive performance are less consistently demonstrated. More standardized and longitudinal studies are needed to identify patients at higher risk and to develop personalized prevention and rehabilitation strategies.

## 1. Introduction

Cancer-related cognitive impairment (CRCI) affects up to 75% of patients with non-CNS cancers, particularly women with breast cancer, as the second most common neoplasm worldwide [1]. CRCI presents as cognitive changes that interfere with daily functioning and quality of life [2,3]. Evidence suggests that roughly one in three cancer survivors experiences clinically significant cognitive impairment [4], with 21–34% showing measurable deficits on standardized neuropsychological tests [4,5]. Domains most frequently affected include planning, processing speed, and attention [6,7,8,9], with downstream effects on perceived health, interpersonal relationships, and work efficiency [10].

Women with breast cancer constitute the group most affected by CRCI [11], which has been linked to oxidative, mitochondrial, and synaptic damage [12] and thus represents a principal target for pharmacological and non-pharmacological interventions. Emotional factors—such as anxiety and intolerance of uncertainty prior to chemotherapy—may amplify inflammation and cognitive impairment [13].

Emerging studies from the literature also explore specialized functions. In musicians, even subtle treatment-related cognitive changes can translate into meaningful performance limitations. In a study conducted within the Mayo Clinic Breast Cancer Registry (*n* = 1871), one-third of participants who identified as musicians reported acute musical toxicity (AMT) during or after treatment, assessed using the Musical Toxicity Questionnaire (MTQ). Chemotherapy was reported as the most impactful treatment (71%), affecting endurance, precision, speed, and technical execution [14].

These observations underscore the salience of patient quality of life in a context where cancer incidence is rising, and survival is improving [15]. Several reviews have previously examined specific aspects of cancer-related cognitive impairment (CRCI), including neurobiological mechanisms, neuropsychological assessment, neuroimaging findings, or individual intervention strategies. However, many of these reviews focused on single domains, including heterogeneous cancer populations, or were conducted in light of the rapid expansion of the recent literature on multimodal rehabilitation, neuroinflammatory mechanisms, and advanced neuroimaging approaches in breast cancer populations.

In addition, substantial heterogeneity persists across studies in terms of cognitive assessment methods, treatment phases, intervention modalities, and outcome measures, making their interpretation and clinical translation challenging.

The present work was, therefore, designed as a structured narrative review aimed at critically synthesizing current evidence on CRCI in breast cancer, with particular focus on neurobiological mechanisms, neuropsychological assessment, neuroimaging correlates, clinical and demographic risk factors, emerging artificial intelligence and machine learning applications, non-pharmacological interventions, long-term outcomes, and preventive strategies. Rather than providing an exhaustive systematic synthesis, the review focuses on specific topic areas selected a priori on the basis of their relevance to the clinical understanding and management of CRCI in breast cancer. Within each domain, the aim was not to provide a comprehensive or in-depth account of all published evidence, but to integrate emerging findings with the previous literature, highlight methodological limitations and inconsistencies, and identify current knowledge gaps and priorities for future research in supportive oncology and survivorship care.

## 2. Materials and Methods

### 2.1. Search Strategy and Eligibility Criteria

To conduct this structured narrative review, a literature search was performed using PubMed/MEDLINE, PsycInfo, and Clinical Key for studies published up to May 2026. In order to retain relevant foundational studies and consensus recommendations, no lower publication-date limit was applied; however, particular emphasis was placed on the literature published between 2023 and 2026.

The search strategy combined keywords and Medical Subject Headings (MeSHs) related to “breast cancer”, “cancer-related cognitive impairment”, “chemobrain”, “chemofog”, “cognitive dysfunction”, “neuroinflammation”, “neuroimaging”, “executive function”, “cognitive rehabilitation”, “psychological interventions”, “mindfulness”, “physical activity”, and “cognitive training”, using Boolean operators (AND/OR) according to database-specific requirements. To address an additional focus on artificial intelligence and machine learning, a targeted supplementary search was conducted in May 2026 using combinations of the terms “artificial intelligence”, “machine learning”, “deep learning”, “speech analysis”, “digital biomarkers”, “breast cancer”, and “cancer-related cognitive impairment”.

Peer-reviewed English-language studies involving adult breast cancer populations and addressing at least one predefined thematic domain of CRCI were considered eligible. Studies focused exclusively on primary central nervous system tumors, pediatric populations, or lacking cognitive-related outcomes were excluded.

### 2.2. Study Selection and Narrative Synthesis

The initial search yielded approximately 100 records across the selected databases. Based on a retrospective reconstruction of the reference library, approximately 35 duplicate or non-eligible records were removed during preliminary screening, and 65 publications were retained for inclusion in the structured narrative synthesis. After preliminary screening and removal of duplicates, the authors reviewed the titles, abstracts, and full-text articles according to their relevance to the predefined thematic domains of the review and their contribution to the current understanding of CRCI in breast cancer. Priority was given to recent systematic reviews, meta-analyses, randomized controlled trials, and large observational studies, while pilot and feasibility studies were included when considered relevant to emerging or exploratory areas of research.

Given the substantial heterogeneity across study designs, interventions, cognitive assessment methods, and outcome measures, a quantitative synthesis was deemed inappropriate. Evidence was therefore synthesized narratively, with attention given to methodological limitations, consistent versus inconsistent findings, type of evidence, and distinctions between subjective and objective cognitive outcomes.

**Figure 1:**
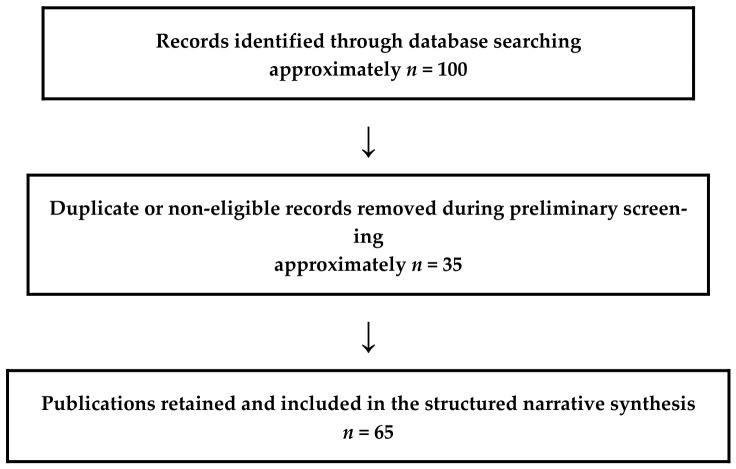
Simplified flow diagram of study selection. The screening counts were reconstructed retrospectively from the reference library; therefore, the initial and exclusion counts are approximate.

As a structured narrative review, no formal risk-of-bias assessment or quantitative evidence grading was performed. A simplified flow diagram of the study selection process is presented in Figure 1.

## 3. Results

The following sections summarize the evidence identified within the predefined thematic domains of this structured narrative review. Findings are presented as an interpretative synthesis, with attention given to consistent and inconsistent findings across studies, methodological limitations, and remaining research gaps.

### 3.1. Neurobiological Mechanisms

The neurobiological mechanisms underlying CRCI are likely multifactorial and only partially established in clinical breast cancer populations. Although several pathways have been proposed, including neuroinflammation, endocrine disruption, oxidative stress, mitochondrial dysfunction, and glial alterations, the strength of evidence varies substantially across mechanisms. In particular, some mechanisms are supported mainly by clinical observational studies, whereas others derive predominantly from preclinical or translational models.

Neuroinflammation is among the most frequently investigated mechanisms of CRCI, and recent clinical findings largely reinforce previous evidence linking inflammatory processes to both objective cognitive performance and subjective cognitive complaints [3,5]. More specifically, increased concentrations of inflammatory cytokines, including IL-1β, IL-6, IL-8, and TNF-α, have been associated with poorer cognitive performance and subjective cognitive complaints in breast cancer patients, sometimes even before the initiation of cancer treatment [16]. During and after chemotherapy, cytokine fluctuations have also been linked to worsening perceived cognition, with some markers showing long-term alterations [17]. However, these associations should not be interpreted as definitive evidence of causality, as inflammation is closely interrelated with fatigue, sleep disturbances, psychological distress, treatment burden, and comorbidities.

Among the endocrine mechanisms examined in breast cancer populations, estrogen deprivation and the cognitive effects of anti-hormonal therapies have received the greatest attention. Estrogen deprivation has been associated with changes in verbal memory and executive functioning, which are domains known to be sensitive to hormonal modulation. Nevertheless, findings remain inconsistent, and the cognitive effects of tamoxifen and aromatase inhibitors are difficult to distinguish from those of age, menopausal status, fatigue, mood symptoms, and prior exposure to chemotherapy [5].

Evidence regarding radiotherapy and immunotherapy remains more limited in breast cancer populations. Radiotherapy may contribute indirectly to cognitive changes through oxidative stress, systemic inflammation, immune dysregulation, and fatigue; however, the evidence for a direct “radio-brain” effect in non-CNS breast cancer remains inconclusive [18]. Similarly, cognitive effects related to immunotherapy appear uncommon; they are mainly described in the context of immune-related neurological adverse events, such as encephalitis or meningoencephalitis, rather than as a well-characterized CRCI phenotype [19,20].

Growing attention has also been directed toward glial mechanisms. Astrocytes, microglia, and oligodendrocytes may contribute to chemotherapy-related neuroinflammation, synaptic dysfunction, altered glutamate homeostasis, and vulnerability of white matter [21,22]. However, much of the evidence supporting glial involvement derives from preclinical models. Astrocytic polarization toward pro-inflammatory phenotypes, microglial activation, and impaired oligodendrocyte maturation provide biologically plausible pathways linking cancer treatments to cognitive dysfunction; however, their direct clinical translation in breast cancer survivors remains to be fully established.

Overall, current evidence continues to support a multifactorial model of CRCI in which inflammatory, endocrine, vascular, psychological, and treatment-related mechanisms interact over time [3,5,11]. However, the relative contribution of each pathway remains uncertain, highlighting the need for longitudinal studies that integrate cognitive assessment, inflammatory biomarkers, endocrine variables, neuroimaging, and patient-reported outcomes.

### 3.2. Neuropsychological Assessment

The neuropsychological assessment of CRCI in breast cancer remains methodologically heterogeneous across studies, particularly regarding test selection, timing of evaluation, and definitions of cognitive impairment. Nevertheless, the recommendations proposed by the International Cognition and Cancer Task Force (ICCTF) continue to represent the principal reference framework for cognitive assessment in oncology settings [23].

The recent literature largely confirms the involvement of domains previously identified as particularly vulnerable in CRCI, including attention, processing speed, executive functioning, working memory, and verbal learning and memory [5,7]. Commonly adopted instruments include the HVLT-R or RAVLT for verbal memory, Trail Making Test A and B for processing speed and cognitive flexibility, and Digit Span, Stroop, and Digit Symbol/Coding tasks for working memory and executive control [6,7,23].

However, current challenges in CRCI assessment extend beyond the identification of specific affected cognitive domains. One of the principal methodological issues remains the limited harmonization of neuropsychological batteries across studies, which contributes substantially to heterogeneity in reported findings and complicates comparisons between populations and interventions [5,7,23].

In parallel, increasing attention has been directed toward the discrepancy frequently observed between objective neuropsychological performance and subjective cognitive complaints. Patient-reported outcomes (PROs), particularly the FACT-Cog, are widely used to capture perceived cognitive functioning and its impact on daily life. Instruments such as PROMIS Cognitive Function and the cognitive subscale of the EORTC QLQ-C30 are also commonly adopted in oncology research and survivorship settings. Although subjective measures are clinically relevant, their correlation with objective cognitive performance remains inconsistent, suggesting that psychological distress, fatigue, sleep disturbances, and emotional burden may contribute to perceived impairment [5,7].

This discrepancy may also reflect differences between the structured, quiet conditions of one-to-one neuropsychological testing and the more complex, distracting demands of everyday home and work environments. Patients may therefore perform adequately on brief standardized tasks while still experiencing clinically meaningful difficulties in daily functioning [6,7].

Global cognitive screening instruments such as the MMSE and MoCA may be useful as preliminary screening tools, particularly in geriatric oncology settings [24]; however, their sensitivity to subtle changes that may be CRCI-related appears limited.

Overall, recent evidence supports the need for more standardized and ecologically valid assessment approaches that integrate objective cognitive testing with patient-reported outcomes and functional measures.

### 3.3. Neuroimaging Techniques

Neuroimaging studies have contributed substantially to the characterization of potential structural, functional, and metabolic correlates of CRCI. However, most available evidence remains research-based, and neuroimaging biomarkers are not yet sufficiently standardized or validated for routine clinical use in breast cancer populations [25,26].

Structural MRI studies have reported reductions in hippocampal, frontal, and temporal volumes, as well as alterations in cortical thickness and gyrification, in association with deficits in memory, attention, and executive functioning. These findings support the hypothesis that CRCI may be associated with measurable brain alterations. Nevertheless, variability in imaging protocols, sample characteristics, treatment exposure, and timing of assessment limits direct comparability across studies [25,26].

Diffusion-weighted imaging and diffusion tensor imaging studies suggest that chemotherapy may be associated with microstructural alterations to white matter, particularly in the corpus callosum, corona radiata, and associative fiber tracts. Reduced fractional anisotropy has been interpreted as a marker of reduced white matter integrity and linked to memory and executive dysfunction. However, these associations are largely correlational and require longitudinal confirmation to clarify whether changes in white matter precede, accompany, or follow cognitive decline [25,26].

Functional MRI studies have provided evidence of alterations in activation and connectivity patterns within networks involved in attention, memory, and executive control. Earlier studies often described compensatory hyperactivation in fronto-parietal regions; however, more recent evidence has reported hypoactivation or altered connectivity involving the hippocampus, precuneus, prefrontal cortex, Default Mode Network, Central Executive Network, and Salience Network. These divergent findings may reflect differences in treatment phase, cognitive task demands, disease stage, compensatory mechanisms, and analytical approaches [25,26].

PET, SPECT, and magnetic resonance spectroscopy studies provide complementary information on perfusion, metabolism, neuroinflammation, and neurochemical alterations. Reported findings include diffuse hypoperfusion, reduced glucose metabolism in prefrontal and limbic regions, possible glial activation assessed through TSPO-PET, and changes in metabolites such as N-acetylaspartate, choline, creatine, and glutamate. Although these techniques are promising for mechanistic research, their clinical interpretation remains limited due to the use of small samples, heterogeneous methods, and a lack of validated diagnostic thresholds [25,26].

Overall, neuroimaging findings support the biological plausibility of CRCI and suggest that structural, functional, and metabolic brain alterations may contribute to cognitive symptoms in breast cancer patients and survivors. Nevertheless, current evidence does not yet support the use of neuroimaging as a routine diagnostic tool for CRCI. Future studies should prioritize longitudinal designs, harmonized imaging protocols, integrated neuropsychological testing and patient-reported outcomes, and validation of imaging biomarkers that predict persistent cognitive impairment.

A summary of the main neurobiological mechanisms and neuroimaging findings discussed above is provided in Table 1.

### 3.4. Clinical-, Demographic- and Treatment-Related Risk Factors

Several clinical, demographic, and psychosocial factors have been associated with increased vulnerability to CRCI in breast cancer populations. However, the relative contribution of individual factors is difficult to determine as cognitive outcomes are influenced by the interaction between patient characteristics, tumor biology, treatment exposure, psychological burden, and comorbid symptoms [5,11,27].

Among demographic factors, older age and lower educational level have been repeatedly associated with poorer cognitive outcomes, likely reflecting reduced cognitive reserve and increased vulnerability to treatment-related stressors. Baseline cognitive performance, comorbidities, menopausal status, and pre-existing fatigue or psychological distress may also influence both objective cognitive performance and perceived cognitive functioning [5,27].

Treatment-related factors are particularly relevant. Chemotherapy remains the treatment modality most consistently associated with CRCI; however, cognitive changes may also occur before treatment initiation, suggesting that cancer-related inflammation, psychological distress, and baseline vulnerability may contribute independently to cognitive changes. Endocrine therapy has been associated with subtle changes in verbal memory and executive functioning in some studies; however, findings remain inconsistent and may be influenced by age, menopausal status, sleep disturbance, fatigue, and prior chemotherapy exposure. Evidence regarding radiotherapy in breast cancer is less consistent, particularly in comparison with central nervous system tumors; it should be considered that systemic inflammatory and fatigue-related pathways may contribute indirectly to CRCI [3,5,11,18].

Tumor-related factors, including pathological subtype, have been associated with CRCI risk, although the evidence remains heterogeneous and largely observational [27]. Treatment intensity and multimodal treatment exposure may also contribute to cognitive burden, but their effects are difficult to disentangle from tumor characteristics and psychosocial stress [5,27].

Although sex-mediated differences have been explored in onco-geriatric populations [20], CRCI studies in breast cancer predominantly include women, and evidence regarding sex- or gender-related differences within breast cancer cohorts remains limited. Future studies should include more diverse cohorts where appropriate and explicitly examine whether sex- or gender-related factors influence CRCI vulnerability, symptom reporting, and recovery trajectories.

A recent study developed a predictive risk model for CRCI in breast cancer patients [27]. In a cohort of 423 women, cognitive impairment was identified in 19.6% of cases based on MoCA scores. Multivariate analysis identified six independent risk factors: advanced age, low educational level, tumor histotype, treatment type, negative emotions, and fatigue. The resulting nomogram showed high discriminative performance in both training and validation sets. Nevertheless, the monocentric and cross-sectional design limits causal interpretation and underscores the need for multicenter longitudinal validation.

Overall, current evidence suggests that CRCI risk is unlikely to be explained by a single demographic, clinical, or treatment-related factor. Rather, CRCI appears to emerge from the cumulative interaction between biological vulnerability, treatment exposure, cancer-related symptoms, and psychological distress [5,27]. Future predictive models should integrate clinical variables, treatment characteristics, baseline cognitive reserve, patient-reported symptoms, biomarkers, and longitudinal cognitive outcomes into their analysis.

### 3.5. Emerging Role of Artificial Intelligence and Machine Learning

Artificial intelligence (AI) and machine learning (ML) approaches are being increasingly explored in oncology to support risk prediction, clinical stratification, imaging interpretation, and personalized care. In the context of CRCI, these methods may be particularly relevant because cognitive outcomes are influenced by multiple interacting variables, including demographic factors, cancer-related characteristics, exposure to treatment, inflammatory and endocrine markers, neuroimaging findings, patient-reported outcomes, fatigue, sleep disturbance, and psychological distress.

Recent reviews on prediction models for the risk of CRCI have highlighted the potential of ML-based approaches to integrate multidimensional clinical and biological data, identify patients at higher risk of cognitive impairment, and guide earlier preventive or rehabilitative strategies [28]. More broadly, AI has been applied across the cancer care continuum in areas such as risk assessment, early detection, treatment planning, supportive care, and survivorship, supporting the general feasibility of computational approaches in oncology [29]. In breast cancer, AI and deep learning have been particularly developed in imaging, including mammography, ultrasound, and MRI, with applications in diagnosis, risk assessment, prognosis prediction, and treatment response monitoring; however, these applications are not CRCI-specific [30].

Emerging CRCI-specific applications include speech-based digital markers to detect subtle subjective cognitive impairment in breast cancer survivors. A recent machine-learning study suggests that speech analysis may provide a fast, ecological, and non-invasive approach to assessing CRCI-related cognitive complaints; however, these findings remain preliminary and require further validation [31].

However, the application of AI to CRCI is in its early stages. Most available studies are exploratory, rely on relatively small or heterogeneous samples, and lack external validation. In addition, AI models may be limited by variability in cognitive endpoints, insufficient integration of objective and subjective outcomes, and reduced interpretability. Therefore, although AI and ML approaches may contribute to future CRCI risk stratification and personalized survivorship care, their clinical implementation requires prospective validation, transparent reporting, and integration with standardized neuropsychological and patient-reported outcome measures [28,29,30,31].

### 3.6. Non-Pharmacological Treatments

The management of CRCI currently relies predominantly on non-pharmacological approaches, although the overall quality and consistency of evidence remain variable across interventions and outcome measures [11]. Nevertheless, several recent studies have explored the potential efficacy of acetylcholinesterase inhibitors (e.g., donepezil) and NMDA receptor antagonists (e.g., memantine), with largely inconclusive results. Trials investigating donepezil have not demonstrated a clear cognitive benefit [32,33], and memantine remains under investigation. Although preliminary findings suggest good tolerability and possible cognitive protection, these results have yet to be confirmed in placebo-controlled studies [34].

This section summarizes recent evidence regarding non-pharmacological interventions for CRCI, with attention given to intervention type, outcome measures, methodological limitations, and consistency of findings. Several recent systematic reviews and meta-analyses suggest that cognitive rehabilitation, physical activity, mindfulness-based interventions, and multimodal supportive approaches may contribute to improvements in cognitive symptoms and quality of life in breast cancer survivors [35,36]. However, findings remain heterogeneous due to substantial variability in study populations, intervention timing and duration, cognitive assessment methods, and reliance on subjective versus objective cognitive outcomes.

In many studies, improvements are more consistently observed in patient-reported cognitive functioning than in standardized neuropsychological performance measures [35,36,37]. This discrepancy represents one of the primary methodological challenges in CRCI research, as it complicates direct comparisons across interventions.

Recent comparative evidence from network meta-analyses suggests that therapist-guided multimodal interventions integrating psychoeducation and structured cognitive rehabilitation may provide broader benefits across cognitive domains compared with single-component interventions [37]. Nevertheless, effect sizes are often modest, heterogeneity remains high in several analyses, and methodological limitations across studies warrant cautious interpretation of the available evidence.

### 3.7. Neuropsychological Approach: Cognitive Training/Cognitive Rehabilitation

Recent evidence suggests that cognitive training and cognitive rehabilitation interventions may contribute to improvements in selected domains of CRCI, particularly executive functioning, processing speed, attention, and perceived cognitive functioning [38,39]. However, the overall evidence remains heterogeneous, with substantial variability in intervention formats, timing, intensity, outcome measures, and methodological quality across studies.

Among the most studied approaches, computerized cognitive training (CCT) has had promising results in several randomized trials. In a recent study by Kleinknecht et al. [40], breast cancer survivors receiving CCT demonstrated reductions in the proportion of participants classified as cognitively impaired, alongside improvements in executive functions and processing speed. A dose–response relationship was also observed, suggesting greater benefits with longer training exposure. Nevertheless, these findings derive from relatively specific study populations and require replication in larger multicenter settings.

Meta-analytic evidence also suggests potential benefits of cognitive training on self-perceived cognitive symptoms. The 2025 network meta-analysis by Yang et al. [35] reported modest improvements in FACT-Cog scores and selected cognitive domains; however, heterogeneity across studies remained substantial, limiting the consistency and generalizability of pooled findings.

Additional exploratory approaches, including eye-tracking-assisted cognitive rehabilitation and biofeedback-based interventions, have been reported to result in preliminary improvements in attentional control and inhibitory processes [41]. Although these findings are of interest from a mechanistic perspective, the available evidence remains limited by small sample sizes and pilot study designs.

Recent systematic reviews focusing specifically on breast cancer populations further support the feasibility and potential clinical relevance of cognitive stimulation interventions [42]. However, one of the principal methodological issues across studies is the discrepancy between subjective and objective cognitive outcomes. Improvements are more consistently observed in self-reported cognitive functioning and perceived cognitive competence than in standardized neuropsychological performance measures, where findings remain more variable and less consistently replicated.

This discrepancy may partly reflect the influence of fatigue, emotional distress, sleep disturbances, and psychological adaptation on perceived cognition, while also highlighting the limited ecological sensitivity of some objective neuropsychological measures commonly used in CRCI research [5,7,35].

Substantial heterogeneity also persists regarding intervention duration, group versus individual delivery, treatment phase, and cognitive assessment methods, further complicating comparisons across studies and limiting the generalizability of findings [35,38,39,42].

Overall, current evidence suggests that cognitive rehabilitation approaches represent promising supportive strategies for CRCI management, particularly when integrated into multimodal survivorship care. Nevertheless, larger standardized trials with longitudinal follow-up and harmonized cognitive endpoints remain necessary to clarify their long-term efficacy and clinical applicability.

### 3.8. Physical Approach: Physical Activity

Physical activity represents one of the most extensively investigated non-pharmacological approaches for CRCI management in breast cancer populations. Recent systematic reviews and meta-analyses suggest that exercise interventions may contribute to improvements in attention, working memory, executive functioning, cognitive fatigue, and perceived cognitive functioning [35,36].

However, the magnitude and consistency of these effects vary substantially across studies depending on exercise modality, intervention intensity, treatment phase, and outcome measures. Improvements are generally more robust for self-reported cognitive outcomes and fatigue-related symptoms than for objective neuropsychological performance measures [35,36].

Meta-analytic findings reported by Yang et al. [35] suggest moderate effects on attentional and executive domains, as well as improvements in perceived cognitive abilities and cognitive complaints. Nevertheless, heterogeneity across interventions and assessment methods remains considerable.

Recent randomized evidence further supports the feasibility and potential clinical relevance of structured exercise during chemotherapy. A multicenter phase III trial demonstrated that a home-based individualized exercise program combining walking and resistance training was associated with reductions in perceived cognitive impairment and mental fatigue compared with standard care [43]. However, benefits were not consistently observed across all chemotherapy schedules or objective cognitive endpoints.

Similarly, the ACTIVATE trial [44], conducted during active chemotherapy, did not demonstrate significant between-group differences in objective cognitive measures after correction for multiple testing; however, improvements in self-reported cognition and quality of life were observed in the exercise group.

Ongoing comparative analyses may further clarify whether specific exercise modalities influence cognitive domains and symptom trajectories differently in breast cancer populations receiving chemotherapy [45].

Overall, current evidence supports physical activity as a promising supportive intervention within survivorship care, particularly for improving perceived cognitive functioning, reducing fatigue, and enhancing quality of life. Nevertheless, substantial heterogeneity across protocols and outcome measures, together with limited consistency of objective cognitive findings, warrants cautious interpretation and highlights the need for standardized longitudinal studies. Beyond CRCI-specific outcomes, physical activity has broader relevance across the oncology continuum, including prevention, survivorship, and functional recovery [46].

### 3.9. Psychological Approach: Mindfulness and Psychological Interventions

Mindfulness-based and psychological interventions have been increasingly investigated as supportive approaches for managing CRCI in breast cancer populations, particularly in relation to perceived cognitive functioning, emotional distress, fatigue, and quality of life [36,47].

Recent network meta-analyses suggest that Acceptance and Commitment Therapy (ACT), mindfulness-based stress reduction (MBSR), meditation-based interventions, and Qigong may be associated with improvements in specific cognitive and psychological domains [35,36]. However, these comparative rankings should be interpreted cautiously, given the substantial heterogeneity across interventions, populations, outcome measures, and study designs.

In several studies, benefits appear more consistent for self-reported cognitive functioning and psychological well-being than for objective neuropsychological performance measures. This pattern suggests that psychological interventions may partially exert their effects through improvements in emotional regulation, stress reduction, fatigue, sleep quality, and coping processes, rather than through direct modification of specific cognitive deficits [35,36,47].

The 2025 network meta-analysis by Yang et al. [35] reported favorable rankings for ACT in global self-perceived cognition, mindfulness-based interventions in processing speed, and physical activity in executive functioning. Nevertheless, the variability of cognitive endpoints and the predominance of subjective outcome measures limit direct comparisons between intervention modalities.

Mechanistic evidence also remains preliminary. An fMRI-based study investigating the CALM psychotherapy intervention reported post-treatment cognitive improvements associated with changes in functional connectivity involving the Default Mode Network and visual networks [48]. Although these findings are of potential neurobiological interest, their clinical significance and reproducibility remain to be established.

Overall, current evidence suggests that mindfulness-based and psychological interventions may represent valuable supportive strategies within multimodal survivorship care, particularly for perceived cognitive impairment and emotional burden. However, further longitudinal studies employing standardized objective cognitive measures and harmonized intervention protocols are needed to clarify their specific cognitive effects and mechanisms of action.

### 3.10. Multimodal Approach: Telemedicine and Digital Methodologies

Digital and remotely delivered interventions have gained increasing attention as potentially scalable approaches for CRCI management in breast cancer populations. These strategies may be particularly relevant in survivorship care, where accessibility, continuity of support, adherence monitoring, and integration of cognitive, physical, and psychological components represent important clinical priorities [49,50].

The Cog-Stim feasibility study by Binarelli et al. [49], which combined computerized cognitive training with adapted physical activity delivered remotely over 12 weeks, reported acceptable feasibility and adherence, with improvements identified in subjective cognition, selected objective cognitive domains, fatigue, and depressive symptoms. However, given the feasibility design, small sample size, and variability in adherence between components, these findings should be interpreted as preliminary rather than definitive evidence of efficacy.

Telemedicine-based psychological interventions, particularly tele-CBT for insomnia, have also shown benefits for sleep and fatigue in breast cancer populations; however, evidence regarding direct cognitive outcomes remains limited and heterogeneous [50]. This distinction is clinically relevant, as improvements in perceived cognition may partly reflect indirect effects mediated by sleep quality, fatigue reduction, mood, and distress rather than direct changes in cognitive performance [49,50].

Emerging multimodal protocols are currently investigating the integration of cognitive training with neuromodulation techniques such as transcranial direct current stimulation (tDCS) [51]. Similarly, stepped-care digital platforms, including the ICOgnition intervention, aim to combine monitoring, psychoeducation, compensatory strategies, mindfulness, and personalized computerized cognitive training within scalable eHealth models [52]. As several of these studies are ongoing, their contribution currently lies primarily in defining feasible and personalized intervention frameworks rather than establishing clinical efficacy.

Overall, multimodal digital approaches appear promising for extending CRCI care beyond traditional in-person settings and for addressing interacting symptoms such as fatigue, sleep disturbance, psychological distress, and perceived cognitive impairment. Nevertheless, larger randomized trials with standardized cognitive endpoints, longer follow-up, and a clear distinction between subjective and objective outcomes are required before these interventions can be considered established therapeutic strategies [49,50,51,52].

Taken together, the available evidence suggests that cognitive training and physical activity are among the most frequently investigated and potentially promising non-pharmacological strategies for CRCI in breast cancer populations [35,36,37,43,44,47,48,49,50,51,52,53]. Cognitive training has been associated with improvements in executive functioning, processing speed, attention, and perceived cognitive competence. By contrast, physical activity appears particularly relevant for reducing fatigue and improving perceived cognitive functioning, attention, and quality of life. Psychological and mindfulness-based interventions may offer additional benefits, particularly for self-reported cognition and emotional distress, while multimodal digital approaches appear feasible and may support more personalized survivorship care.

However, comparative conclusions across intervention types should be interpreted cautiously. Existing evidence remains heterogeneous with respect to study design, intervention dose and timing, treatment phase, and cognitive endpoints. In particular, improvements are more consistently reported for patient-reported cognitive outcomes than for objective neuropsychological measures. Therefore, although non-pharmacological interventions represent promising supportive strategies, their relative efficacy, optimal delivery format, and long-term impact remain insufficiently established.

The main non-pharmacological interventions, their cognitive relevance, and key limitations are summarized in Table 2.

In addition to published evidence, several active or ongoing randomized trials are currently investigating cognitive training, physical activity, pharmacological approaches, and neuromodulation strategies for CRCI in breast cancer. These trials are summarized in Table 3.

### 3.11. Long-Term Outcomes

Long-term trajectories of CRCI in breast cancer populations remain heterogeneous. In many patients, cognitive symptoms appear to peak during treatment or within the first months after chemotherapy is initiated and may partially improve over the following 1–2 years [5,54,55]. However, a clinically relevant subset of patients continues to report persistent cognitive difficulties or demonstrate measurable deficits over longer follow-up periods [4,56].

The persistence of CRCI is likely to be influenced by multiple interacting factors. These include baseline cognitive reserve, age, comorbidities, treatment exposure, endocrine status, fatigue, sleep disturbances, psychological distress, and inflammatory burden. This complexity makes it difficult to distinguish treatment-related cognitive decline from pre-existing vulnerability, changes related to aging, and cancer-related systemic effects [5,11,27,54,55,56,57,58].

Neuroimaging studies have reported associations between exposure to breast cancer treatment and structural brain alterations, including reductions in cortical thickness or regional brain volumes for areas involved in memory, executive functioning, and attentional control [57]. Some findings suggest that tumor subtype and receptor status may be associated with differential patterns of cortical vulnerability. Nevertheless, these associations remain largely observational, and causal pathways linking a specific tumor biology, treatment exposure, brain atrophy, and long-term cognitive decline require further clarification.

Concerns have also been raised regarding a possible association between CRCI and neurodegenerative risk later in life [58]. However, current evidence does not draw firm conclusions regarding whether CRCI represents an independent risk factor for dementia, an acceleration of pre-existing vulnerability, or a transient treatment-related phenomenon in most patients. Therefore, the overlap between CRCI-related neuroimaging patterns and those described in neurodegenerative or vascular cognitive disorders should be interpreted cautiously.

Overall, long-term CRCI should be considered a heterogeneous clinical phenomenon with variable recovery trajectories. Future studies should employ longitudinal designs with baseline pre-treatment assessment, repeated neuropsychological testing, patient-reported outcomes, biomarkers, and advanced neuroimaging to identify patients at risk for persistent impairment and distinguish transient cognitive symptoms from progressive cognitive decline [4,5,54,55,56,57,58].

### 3.12. Preventive Strategies

Preventive strategies for CRCI in breast cancer remain insufficiently established, and no pharmacological intervention has yet demonstrated consistent preventive efficacy in adults with non-CNS cancers. Current evidence, therefore, supports a focus on early supportive and behavioral interventions aimed at reducing cognitive vulnerability and mitigating modifiable contributors to perceived and objective cognitive decline [44,59,60,61,62,63].

Physical activity initiated during chemotherapy, sleep-focused interventions such as cognitive behavioral therapy for insomnia, mind–body practices, and early cognitive training have all shown potential benefits on perceived cognitive functioning, fatigue, sleep quality, psychological distress, and quality of life [44,59,60,61,62,63]. However, these approaches should be interpreted as early supportive or risk-mitigation strategies at present rather than established preventive treatments.

A key limitation of the current evidence is that improvements are more consistently reported for patient-reported cognitive outcomes and associated symptoms than for objective neuropsychological performance. This discrepancy makes it difficult to determine whether these interventions directly prevent cognitive decline or indirectly improve perceived cognition through effects on sleep, fatigue, mood, and coping processes.

**Figure 2:**
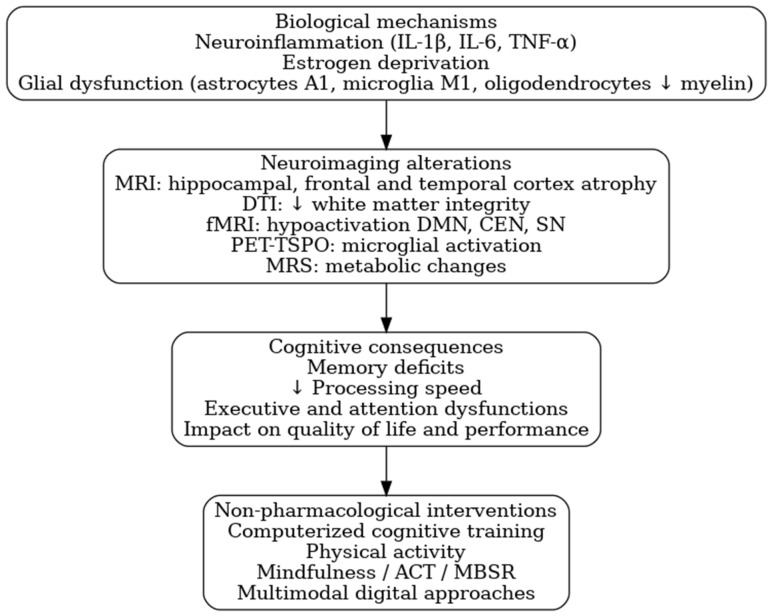
Proposed multifactorial mechanisms contributing to CRCI in breast cancer. The figure illustrates the interaction between treatment-related factors, neuroinflammatory and endocrine pathways, alterations in glial and white matter, psychological symptoms, sleep disturbance, fatigue, and cognitive outcomes. These pathways should be interpreted as interrelated mechanisms with varying levels of clinical and preclinical evidence.

Overall, prevention-oriented research should clearly distinguish between prevention of objective cognitive decline, reductions in subjective cognitive complaints, and improvement in associated symptoms. Future trials should include baseline pre-treatment cognitive assessments, standardized endpoints, longer follow-up periods, and stratification of patients by treatment exposure and clinical risk factors. The complex interplay between these neurobiological, psychological, and behavioral factors is illustrated in Figure 2, which summarizes the principal mechanisms underlying CRCI in breast cancer.

To further synthesize the evidence across domains, Table 4 summarizes the main evidence type, overall interpretation, and remaining gaps for each thematic area of this review.

## 4. Discussion

This structured narrative review highlights that CRCI in breast cancer is best understood as a multifactorial and heterogeneous condition rather than a direct and uniform consequence of chemotherapy alone. The current literature supports a model involving interacting neurobiological, treatment-related, psychological, and behavioral mechanisms, including inflammation, endocrine disruption, fatigue, sleep disturbance, emotional distress, and individual differences in cognitive reserve.

However, the strength of evidence varies substantially across domains, and many findings remain limited by heterogeneous study designs, variable assessment methods, small samples, and inconsistent differentiation between subjective and objective cognitive outcomes.

A central methodological issue emerging from the literature is the marked heterogeneity in study designs, assessment tools, outcome measures, and timing of evaluation. CRCI has been operationalized differently across studies, with variable reliance on objective neuropsychological tests, patient-reported outcomes, or a combination of both. Assessments range from active treatment to assessments several years after completion, making direct comparisons difficult and partly explaining inconsistencies across studies, particularly regarding endocrine therapy, neuroimaging correlates, and non-pharmacological interventions.

From a mechanistic perspective, neuroinflammation, endocrine disruption, oxidative stress, and glial alterations represent biologically plausible pathways linking breast cancer and its treatments to cognitive changes. Nevertheless, these mechanisms should be interpreted with different levels of evidentiary strength. Inflammatory markers have been associated with cognitive complaints and performance in clinical studies [16,17]. By contrast, findings of glial activation, astrocytic polarization, and oligodendrocyte vulnerability are largely derived from preclinical and translational evidence [21,22,64,65]. This distinction is important to avoid extending mechanistic hypotheses beyond their current clinical validation. Similarly, estrogen deprivation resulting from endocrine therapy may contribute to changes in verbal memory and executive functioning; however, findings remain inconsistent and are difficult to distinguish from the effects of menopausal status, fatigue, sleep disturbance, mood symptoms, and previous chemotherapy exposure [5].

Neuroimaging findings provide complementary evidence supporting the biological plausibility of CRCI, including structural, functional, and metabolic alterations involving regions and networks implicated in memory, attention, and executive control [25,26]. However, these findings remain largely research-based and are not yet sufficiently standardized for routine clinical use. Reported changes in hippocampal and fronto-temporal volumes, white matter integrity, functional connectivity, and metabolic activity should therefore be interpreted as correlates of CRCI rather than validated diagnostic or predictive biomarkers. PET-based markers of neuroinflammation and glial activation are particularly promising for mechanistic research, but their clinical role in breast cancer survivors remains preliminary.

A second major issue concerns the distinction between subjective cognitive complaints and objective neuropsychological performance. Patient-reported outcomes are clinically meaningful because they capture perceived cognitive difficulties and their impact on daily functioning, quality of life, occupational efficiency, and social participation [10]. However, subjective cognitive impairment is also influenced by fatigue, sleep disturbance, anxiety, depression, and illness-related stress. Conversely, standardized neuropsychological tests may lack ecological sensitivity to subtle real-world difficulties [6,7].

Future studies should therefore integrate both approaches rather than treating them as interchangeable endpoints.

The evidence for non-pharmacological interventions is encouraging but not definitive. Cognitive training, physical activity, mindfulness-based approaches, psychological interventions, and multimodal digital programs appear feasible and may improve perceived cognitive functioning, fatigue, emotional distress, quality of life, and specific cognitive domains [35,36,40,44,47,48,49]. However, the available evidence remains heterogeneous, and improvements are generally more consistent for patient-reported outcomes than for objective neuropsychological measures. Comparative evidence, including network meta-analyses, suggests that therapist-guided multimodal programs integrating psychoeducation and structured cognitive rehabilitation may offer broader benefits than single-component interventions [37]. Nevertheless, such comparisons should be interpreted cautiously as intervention content, dose, timing, and endpoints vary substantially across studies.

Therefore, non-pharmacological interventions should currently be considered promising supportive strategies rather than established therapeutic standards for CRCI. Their potential clinical value may lie not only in direct cognitive effects but also in the modulation of fatigue, sleep quality, emotional regulation, stress-related processes, coping, and perceived cognitive self-efficacy. This broader interpretation is particularly relevant for psychological and mindfulness-based interventions, the effects of which may be mediated by improvements in emotional burden and attentional resources rather than by direct modification of disease-specific neurobiological pathways.

Longitudinal evidence suggests that CRCI symptoms may attenuate within 1–2 years for many patients, although a subset continues to experience persistent cognitive difficulties or measurable deficits over longer periods [4,5,55,56]. Associations between breast cancer, treatment exposure, cortical atrophy, and later-life neurodegenerative risk have been reported [57,58], but causal pathways remain unclear. These findings should therefore be interpreted as signals requiring further investigation rather than evidence that CRCI necessarily represents a progressive neurodegenerative process.

Emerging computational approaches, including artificial intelligence and machine learning, may contribute to future risk stratification and personalized survivorship care by integrating clinical, cognitive, neuroimaging, biomarker, and patient-reported data. However, most applications remain exploratory and require external validation, transparent reporting, and prospective evaluation before they can inform clinical decision-making.

Taken together, these findings highlight the need for integrated and personalized approaches to CRCI that address its multifactorial nature across biological, psychological, and behavioral domains.

Future research should prioritize longitudinal, adequately powered studies that begin before treatment initiation and incorporate repeated cognitive assessments, patient-reported outcomes, biomarkers, neuroimaging, and detailed treatment characterization. Future progress will also require greater consensus on the operational definition of CRCI, harmonized cognitive endpoints, standardized intervention protocols, and stratification by clinical and demographic risk factors. Multisite collaborative studies with adequately powered and more diverse samples will be essential to improve reproducibility and generalizability, identify patients most likely to experience persistent CRCI, and determine which interventions are most appropriate for which patients, at what time point, and through which mechanisms.

### 4.1. Clinical and Research Implications

From a clinical perspective, CRCI requires early recognition and multidisciplinary management within survivorship care. Routine screening should combine patient-reported cognitive complaints with targeted objective assessments when clinically indicated, while also considering the effects of fatigue, sleep disturbance, emotional distress, menopausal symptoms, comorbidities, and treatment history.

Accessible non-pharmacological interventions, including cognitive rehabilitation, physical activity, psychological support, sleep-focused interventions, and mindfulness-based approaches, may be considered supportive strategies tailored to the patient’s symptom profile and risk factors. However, these interventions should be presented clinically as supportive and potentially beneficial rather than as definitive treatments for CRCI.

From a research perspective, future studies should adopt standardized cognitive batteries, clearly distinguish subjective and objective endpoints, define the intervention dose and timing, and include longer follow-up periods. Integrating neuroimaging, inflammatory and endocrine biomarkers, digital measures, and computational models may lead to improvements in risk prediction and help identify patient subgroups most likely to benefit from specific interventions.

### 4.2. Limitations

This review has several limitations. The review was deliberately limited in scope and was not designed to provide an exhaustive assessment of every domain related to CRCI. Its purpose was to summarize selected areas of the current literature and to integrate recent findings with established knowledge.

First, it was designed as a structured narrative review rather than a systematic review or meta-analysis. Although the search strategy and selection process were made explicit, no formal systematic review protocol, risk-of-bias assessment, or quantitative evidence grading was applied. Therefore, selection bias cannot be excluded, and conclusions should be interpreted as an integrative synthesis rather than a definitive quantitative assessment of efficacy.

Second, the literature search was limited to three databases, English-language publications, and studies published up to May 2026, with particular emphasis on the literature published between 2023 and 2026. Relevant studies published in other languages or indexed in other databases may therefore have been missed. Although no lower publication-date limit was applied, particular emphasis was placed on the literature published between 2023 and 2026; consequently, the older literature may have been represented less comprehensively.

Third, the included literature is highly heterogeneous in terms of study design, sample size, cancer stage, treatment exposure, timing of assessment, intervention type, and outcome measures. Several cited studies are pilot, feasibility, or single-center trials with limited sample sizes and short follow-ups. These limitations restrict direct comparability across studies and reduce the certainty of conclusions regarding the efficacy of interventions.

Finally, the multifactorial nature of CRCI means that it is difficult to establish causal relationships between specific cancer treatments, biological mechanisms, psychological factors, and cognitive outcomes. In particular, the frequent discrepancy between subjective cognitive complaints and objective neuropsychological performance complicates interpretation and highlights the need for harmonized assessment strategies in future research.

## 5. Conclusions

CRCI in breast cancer represents a multifactorial and heterogeneous condition in which neurobiological, treatment-related, psychological, and behavioral factors interact over time. Current evidence implicates inflammatory, endocrine, oxidative, and glial pathways in this condition, together with structural and functional brain alterations identified through neuroimaging studies. However, the relative contribution of these mechanisms remains incompletely defined, and many findings require further longitudinal and clinically validated investigation.

Non-pharmacological interventions, including cognitive training, physical activity, mindfulness-based and psychological approaches, and multimodal digital programs, appear promising as supportive strategies for CRCI management. Nevertheless, available evidence remains heterogeneous, with benefits more consistently reported for patient-reported cognitive outcomes, fatigue, emotional distress, and quality of life than for objective neuropsychological performance. These interventions should therefore be considered potentially useful components of personalized survivorship care rather than established definitive treatments.

Future research should prioritize longitudinal studies that begin before treatment initiation; standardized cognitive assessment protocols; a clearer distinction between subjective and objective outcomes; and integration of biomarkers, neuroimaging, digital tools, and computational approaches. Such efforts may lead to improvements in risk stratification, facilitate early identification of vulnerable patients, and guide more individualized prevention and rehabilitation strategies.

In summary, CRCI should no longer be regarded as a transient or nonspecific side effect of breast cancer treatment. It requires early recognition, careful clinical monitoring, and multidisciplinary supportive care aimed at preserving cognitive function, daily functioning, and long-term quality of life.

## Figures and Tables

**Table 1:** Current evidence on neurobiological mechanisms and neuroimaging findings in cancer-related cognitive impairment (CRCI).

Mechanism/Technique	Main Findings	Cognitive Relevance	Evidence Status/Interpretation	References
Neuroinflammation	Elevated inflammatory cytokines (IL-1β, IL-6, IL-8, and TNF-α) have been associated with poorer cognitive performance and subjective cognitive complaints.	May contribute to cognitive vulnerability and perceived impairment.	Clinical observational evidence; associations should not be interpreted as definitive causality.	[16,17]
Endocrine factors	Estrogen deprivation during endocrine therapy and variable cognitive findings with tamoxifen/aromatase inhibitors have been described.	May affect verbal memory and executive functioning, with indirect effects through fatigue, sleep, and mood.	Clinical evidence is inconsistent; effects are difficult to distinguish from age, menopause, fatigue, and prior exposure to chemotherapy.	[5]
Radiotherapy	Potential immune dysregulation, oxidative stress, fatigue-related pathways, and systemic inflammatory effects have been described.	Possible indirect contribution to cognitive complaints.	Evidence in non-CNS breast cancer remains limited and inconclusive.	[18]
Immunotherapy	Cognitive effects have been mainly described in relation to immune-related neurological adverse events.	Not yet characterized as a typical CRCI phenotype in breast cancer.	Early-stage evidence; direct cognitive effects remain insufficiently established.	[19,20]
Glial cells—Astrocytes	Astrocytic activation and pro-inflammatory polarization have been described in treatment-related models.	May contribute to altered glutamate homeostasis, synaptic dysfunction, and reduced plasticity.	Mainly preclinical/translational evidence; clinical relevance remains to be established.	[21,22]
Glial cells—Microglia	Microglial activation and increased pro-inflammatory signaling have been reported in experimental models.	May contribute to neuroinflammatory processes and synaptic vulnerability.	Predominantly preclinical evidence; not yet validated as a clinical biomarker of CRCI.	[21,22]
Glial cells—Oligodendrocytes	Impaired oligodendrocyte maturation and myelin-related alterations have been reported in preclinical models.	May contribute to white matter vulnerability and attentional or memory-related difficulties.	Biologically plausible pathway; clinical translation remains preliminary.	[21]
Volumetric MRI	Reduced hippocampal, frontal, or temporal volumes and cortical thickness/gyrification changes have been reported.	Associated with memory, attention, and executive function complaints or performance.	Research-based evidence; variability in protocols, samples, and comparability of timing limits.	[25,26]
DWI/DTI	White matter microstructural alterations, including corpus callosum, corona radiata, and associative tracts, have been described.	Associated with memory and executive functioning in some studies.	Correlational evidence; longitudinal studies are needed to clarify temporal relationships.	[25,26]
fMRI (BOLD)	Altered activation or connectivity in fronto-parietal regions, hippocampus, precuneus, prefrontal cortex, DMN, CEN, and SN have been reported.	May reflect compensatory or disrupted network functioning in attention, memory, and executive control.	Findings vary by task, treatment phase, analytic approach, and sample characteristics.	[25,26]
PET/SPECT/MRS	Perfusion, glucose metabolism, TSPO-PET neuroinflammation signals, and metabolite changes (NAA, choline, creatine, and glutamate) have been reported.	May provide complementary markers of metabolic or neurochemical vulnerability.	Promising for mechanistic research; not validated for routine diagnosis or prediction.	[25,26]

Note. Evidence categories are descriptive and do not represent formal GRADE ratings. Mechanistic evidence includes clinical observational, preclinical, and translational findings with varying levels of certainty. Abbreviations: BOLD, blood oxygen level-dependent; CEN, Central Executive Network; CRCI, cancer-related cognitive impairment; DMN, Default Mode Network; DTI, diffusion tensor imaging; DWI, diffusion-weighted imaging; fMRI, functional magnetic resonance imaging; GRADE, Grading of Recommendations Assessment, Development and Evaluation; IL, interleukin; MRI, magnetic resonance imaging; MRS, magnetic resonance spectroscopy; NAA, N-acetylaspartate; PET, positron emission tomography; SN, Salience Network; SPECT, single-photon emission computed tomography; TNF-α, tumor necrosis factor-alpha; TSPO-PET, translocator protein positron emission tomography.

**Table 2:** Non-pharmacological interventions and cognitive outcomes in cancer-related cognitive impairment (CRCI).

Intervention Type	Main Findings	Cognitive Relevance	Key Limitations	References
Cognitive training/rehabilitation	Randomized trials and meta-analyses suggest potential benefits, particularly for perceived cognitive functioning and selected domains such as executive functioning, processing speed, and attention. Some studies report dose–response effects.	May support executive functioning, processing speed, attention, and perceived cognitive competence.	Protocols, duration, delivery format, and outcome measures vary substantially. Objective cognitive effects are less consistently replicated than patient-reported improvements.	[35,38,39,40,41,42]
Physical exercise	Exercise interventions have been associated with improvements in fatigue, quality of life, perceived cognitive functioning, and selected attentional or executive outcomes.	May be particularly relevant for cognitive fatigue, perceived cognition, attention, and functional recovery.	Effects vary by modality, intensity, timing, and treatment phase. Objective neuropsychological benefits remain inconsistent across trials.	[35,36,43,44,45,46]
Mindfulness/psychological interventions	ACT, mindfulness-based interventions, meditation, and Qigong have shown promising effects on self-reported cognition levels, emotional distress, coping, and quality of life.	May improve perceived cognitive functioning indirectly through effects on stress, fatigue, sleep, emotional regulation, and attentional resources.	Comparative rankings from network meta-analyses should be interpreted cautiously. Objective cognitive outcomes are heterogeneous, and mechanisms remain incompletely defined.	[35,36,47,48]
Multimodal/digital programs	Tele-CCT, physical activity, eHealth stepped-care models, and combined approaches appear feasible and acceptable, with preliminary evidence of multidomain benefits.	May support scalable and personalized survivorship care by integrating cognitive, physical, and psychological components.	Evidence is mainly preliminary- or feasibility-based. Larger randomized trials with standardized cognitive endpoints and longer follow-up are needed.	[49,50,51,52,53]
Overall interpretation	Cognitive training and physical activity are among the most frequently investigated approaches, while psychological and multimodal interventions may provide additional supportive benefits.	Non-pharmacological interventions appear promising as supportive strategies, especially for patient-reported outcomes, fatigue, distress, and quality of life.	Relative efficacy, optimal timing, delivery format, dose, and long-term impact remain insufficiently established.	[35,37,42,53]

Abbreviations: ACT, Acceptance and Commitment Therapy; CCT, computerized cognitive training; CRCI, cancer-related cognitive impairment; eHealth, electronic health.

**Table 3:** Randomized clinical trials on CRCI in breast cancer (active or ongoing). Trial status and details should be interpreted as current at the date of the ClinicalTrials.gov search.

Trial/Identifier	Study Focus	Design/Status	Population	Intervention/Comparator	Main Cognitive Outcome
NRG-CC011/NCT05896189	Computerized cognitive training for CRCI in breast cancer survivors	Phase III, multicenter, randomized, double-blind controlled trial	Stage I–III breast cancer survivors, 6–60 months post-treatment, with CRCI	Computerized cognitive training (BrainHQ); comparison of training methods	Perceived cognitive impairment (FACT-Cog) and objective cognitive testing
NCT06686823	Cognitive training during active treatment (CHEMOBRAIN MAMA)	Randomized, two-arm, parallel-group trial	Newly diagnosed breast cancer patients receiving active treatment	Personalized cognitive training (everyday cognition) versus control/usual care	MoCA and patient-reported cognitive outcomes, including FACT-Cog
NCT07165912	Group versus individual cognitive training during chemotherapy	Randomized, parallel-group trial	Breast cancer patients undergoing active treatment	Computerized cognitive training for groups versus individuals	Cognitive function and CRCI symptoms
NCT07017530	Exercise combined with computerized cognitive training	Randomized controlled trial	Women with breast cancer and cognitive complaints	Exercise plus computerized cognitive training	Executive function and broader cognitive endpoints
NCT06727773	Memantine and exercise during chemotherapy	Randomized, three-arm, placebo-controlled trial	Stage I–III breast cancer patients receiving chemotherapy with mild cognitive difficulties	Memantine plus exercise versus memantine alone versus placebo/control condition	Cognitive function and CRCI-related biomarkers
NCT07112521	Home-based tDCS for CRCI in breast cancer survivors	Randomized, sham-controlled trial	Breast cancer survivors with CRCI	Active transcranial direct current stimulation plus cognitive tasks versus sham/control trial	Change in cognitive function and CRCI symptoms
NCT04669301 (MAAT-G)	Memory and Attention Adaptation Training—Geriatrics	Phase II randomized pilot trial	Older adults with breast cancer receiving chemotherapy	MAAT-G compensatory cognitive rehabilitation versus control/usual care	Cognitive function, including patient-reported cognition (e.g., FACT-Cog)

Abbreviations: CRCI, cancer-related cognitive impairment; FACT-Cog, Functional Assessment of Cancer Therapy–Cognitive Function; MAAT-G, Memory and Attention Adaptation Training–Geriatrics; MoCA, Montreal Cognitive Assessment; tDCS, transcranial direct current stimulation.

**Table 4:** Summary of evidence type, overall interpretation, and remaining gaps across CRCI domains.

Domain	Main Evidence	Overall Interpretation	Key Limitations/Gaps
Neurobiological mechanisms	Clinical observational + preclinical/translational studies	Multifactorial model; inflammation and endocrine pathways clinically relevant; glial mechanisms biologically plausible	Causality unclear; glial evidence mainly preclinical; need longitudinal biomarker studies
Neuropsychological assessment	Consensus recommendations + observational studies	ICCTF core domains remain the reference framework	Poor harmonization; limited ecological validity; subjective/objective discrepancy
Neuroimaging	MRI, DTI, fMRI, and PET/SPECT/MRS studies	Structural, functional, and metabolic correlates support biological plausibility	Small samples; heterogeneous protocols; not ready for routine clinical use
Risk factors	Observational studies + prediction models	Age, education, treatment exposure, fatigue, distress, and tumor features may influence risk	Mostly observational; limited external validation; causal pathways unclear
AI/ML approaches	Exploratory computational studies	Promising for risk stratification and digital phenotyping	Early-stage evidence; small samples; lack of external validation
Cognitive training	RCTs + meta-analyses	Promising, especially for perceived cognition and selected domains	Heterogeneous protocols; objective effects less consistent
Physical activity	RCTs + meta-analyses	Promising for fatigue, QoL, perceived cognition, and attention	Objective cognitive effects variable; intervention dose unclear
Mindfulness/psychological interventions	RCTs + network meta-analyses	May improve perceived cognition, distress, coping, and QoL	Effects may be indirect; comparative rankings need caution
Digital/multimodal interventions	Feasibility studies + ongoing RCTs	Scalable and feasible; may integrate cognitive, physical, and psychological care	Mostly preliminary; efficacy not established
Long-term outcomes	Longitudinal observational studies	Many patients improve over 1–2 years; a subset has persistent difficulties	Long-term trajectories unclear; dementia links not causal
Preventive strategies	Early intervention studies	Best understood as risk mitigation, not established prevention	Need pre-treatment baseline, longer follow-up, standardized endpoints

Note. Evidence categories are descriptive and do not represent formal GRADE ratings. AI, artificial intelligence; CRCI, cancer-related cognitive impairment; DTI, diffusion tensor imaging; fMRI, functional magnetic resonance imaging; ICCTF, International Cognition and Cancer Task Force; ML, machine learning; MRI, magnetic resonance imaging; MRS, magnetic resonance spectroscopy; PET, positron emission tomography; QoL, quality of life; RCT, randomized controlled trial; SPECT, single-photon emission computed tomography.

## Data Availability

No new data were created or analyzed in this study. Data sharing is not applicable to this article.

## Abbreviations

The following abbreviations are used in this manuscript:

**Abbreviation**	**Full term**
ACT	Acceptance and Commitment Therapy
AI	Artificial Intelligence
AMT	Acute Musical Toxicity
AUC	Area Under the Curve
BOLD	Blood Oxygen Level-Dependent
CALM	Managing Cancer and Living Meaningfully
CBT-I	Cognitive Behavioral Therapy for Insomnia
CCT	Computerized Cognitive Training
CEN	Central Executive Network
CNS	Central Nervous System
CRCI	Cancer-Related Cognitive Impairment
CYP	Cyclophosphamide
DMN	Default Mode Network
DOX	Doxorubicin
DTI	Diffusion Tensor Imaging
DWI	Diffusion-Weighted Imaging
eHealth	Electronic Health
EORTC QLQ-C30	European Organisation for Research and Treatment of Cancer Quality of Life Questionnaire Core 30
EPI	Epirubicin
FACT-Cog	Functional Assessment of Cancer Therapy—Cognitive Function
FDG-PET	Fluorodeoxyglucose Positron Emission Tomography
fMRI	Functional Magnetic Resonance Imaging
GFAP	Glial Fibrillary Acidic Protein
GRADE	Grading of Recommendations Assessment, Development and Evaluation
HVLT-R	Hopkins Verbal Learning Test—Revised
ICCTF	International Cognition and Cancer Task Force
IL	Interleukin
MAAT-G	Memory and Attention Adaptation Training—Geriatrics
MBP	Myelin Basic Protein
MBSR	Mindfulness-Based Stress Reduction
MeSH	Medical Subject Headings
ML	Machine Learning
MMSE	Mini-Mental State Examination
MoCA	Montreal Cognitive Assessment
MRI	Magnetic Resonance Imaging
MRS	Magnetic Resonance Spectroscopy
MTQ	Musical Toxicity Questionnaire
NAA	N-acetylaspartate
NMA	Network Meta-Analysis
NMDA	N-methyl-D-aspartate
OPC	Oligodendrocyte Progenitor Cell
PET	Positron Emission Tomography
PRO	Patient-Reported Outcome
PROMIS	Patient-Reported Outcomes Measurement Information System
QoL	Quality of Life
RAVLT	Rey Auditory Verbal Learning Test
RCT	Randomized Controlled Trial
SMD	Standardized Mean Difference
SN	Salience Network
SPECT	Single-Photon Emission Computed Tomography
SUCRA	Surface Under the Cumulative Ranking Curve
tDCS	transcranial Direct Current Stimulation
TNF-α	Tumor Necrosis Factor-Alpha
TSPO-PET	Translocator Protein Positron Emission Tomography
